# Changes of High-Purity Insoluble Fiber from Soybean Dregs (Okara) after Being Fermented by Colonic Flora and Its Adsorption Capacity

**DOI:** 10.3390/foods10102485

**Published:** 2021-10-17

**Authors:** Bo Lyu, Yi Wang, Xin Zhang, Yuxi Chen, Hongling Fu, Tong Liu, Jianyu Hao, Yang Li, Hansong Yu, Lianzhou Jiang

**Affiliations:** 1 College of Food Science, Northeast Agricultural University, Harbin 150030, China; michael_lvbo@163.com (B.L.); 18249032236@163.com (X.Z.); yangli@neau.edu.cn (Y.L.); 2 Soybean Research & Development Center, Division of Soybean Processing, Chinese Agricultural Research System, Changchun 130118, China; Wangyi284419@163.com (Y.W.); chenyuxi19970624@163.com (Y.C.); 15764381475@163.com (H.F.); 3 College of Food Science and Engineering, Jilin Agricultural University, Changchun 130118, China; 4 College of Food Science and Engineering, Changchun University, Changchun 130118, China; liut@ccu.edu.cn; 5 School of Food and Biotechnology, Changchun Vocational Institute of Technology, Changchun 130118, China; haojianyu55@126.com

**Keywords:** soybean residue, insoluble dietary fiber, gut microbiota, fermentation in vitro, colon health

## Abstract

In order to explore the changes and properties of high-purity insoluble dietary fiber from okara (HPIDF) after entering the colon and be fermented by colonic flora, fermented high-purity insoluble dietary fiber (F-HPIDF) was obtained by simulated fermentation in vitro by HPIDF and colonic flora from C57BL/6 mice. For exploring the differences of HPIDF and F-HPIDF, the changes of structure (SEM. FTIR, XRD, particle size, specific surface area, monosaccharide composition) and adsorption properties (water, oil, heavy metal irons, harmful substances) of HPIDF/F-HPIDF were explored. The results showed that F-HPIDF had a higher water-holding capacity (19.17 g/g), water-swelling capacity (24.83 mL/g), heavy metals-adsorption capacity (Cd^2+^: 1.82 μmol/g; Pb^2+^: 1.91 μmol/g; Zn^2+^: 1.30 μmol/g; Cu^2+^: 0.68 μmol/g), and harmful substances-adsorption capacity (GAC: 0.23 g/g; CAC: 14.80 mg/g; SCAC: 0.49 g/g) than HPIDF due to the changes of structure caused by fermentation. In addition, with the fermentation of HPIDF, some beneficial substances were produced, which might be potential intestinal prebiotics. The study of F-HPIDF strengthens the speculation that HPIDF may have potential bioactivities after entering the colon, which proved that okara-HPIDF may have potential functionality.

## 1. Introduction

The functionality of insoluble dietary fiber (IDF) has been gradually accepted by consumers [1,2,3]. Especially for intestinal health, IDF may have unique physiological activities [4,5,6]. As a kind of material that can improve intestinal health, IDF had been gradually developed as a kind of functional food material. However, recent studies have shown that some kinds of IDF are not necessarily beneficial to intestinal health [7,8,9], which may be due to the complex fermentation mode of IDF after entering the colon. It had been proved that roughly ten subtypes of dietary fibers described to date, categorized as soluble or insoluble, with varying chemical structures, and large differences in their fermentation profiles [10]. This means that the changes of gut microbiota and colonic environment are complex. Therefore, the changes of different kinds of IDF in the colon become worthy to study.

Okara (Soybean dregs), the main by-product during traditional soybean products processing and the production of soybean protein isolate (SPI), had caused huge environmental pollution and waste of resources [11], which includes a large amount of dietary fiber (DF). Okara-DF had been proved to be functional, such as decreased content of blood glucose [12], protected the intestine [13], used as a food raw material ingredient [14], etc. However, different from soluble dietary fiber (SDF), the application of Okara-IDF had a core problem—IDF was difficult to purify. The traditional processing method of IDF is often accompanied by a large amount of protein and soluble fiber as remains. It is difficult to evaluate the functionality of one component in the mixture, as while as caused a disturbance, In addition, okara was difficult to dry because of its unique structure [15]. For these reasons, the usage or value-increment of Okara-IDF as a material of functional food was restricted.

To solve the problem, we prepared high purity insoluble dietary fiber from okara (Okara-HPIDF) by enzymatic method, which purity could exceed 90% [16], and proved that it had potential bioactivities. Meanwhile, we proved that Okara-HPIDF had certain functions, such as improving lipid metabolism and so on [17,18]. In the process of studying its relationship with the changes of the colonic environment, we found that Okara-HPIDF could significantly change the composition of colonic flora [18]. This means that Okara-HPIDF was fully fermented by microorganisms in the colon, which may lead to changes in structure, composition, functionality, and even bioactivities of Okara-HPIDF. The above changes had not been studied, because we used germ-free simulated digestive fluid (SGF) in the previous study [16].

In this way, this study aimed to explore the changes after Okara-HPIDF enters the colon, while being fermented by colonic microorganisms. In this work, we were committed to treating Okara-HPIDF by simulated intestinal fluid (SIF) with colonic microorganisms and digestive enzymes, to look for the changes of Okara-HPIDF after fermentation. The structure, monosaccharide composition, and physicochemical properties before and after fermentation were studied, and a more accurate model of simulated fermentation in vitro of Okara-HPIDF was established. This study is a supplement to our team’s previous research, to explore the changes and properties of HPIDF after entering the colon and be fermented by colonic flora, which will improve our evaluation system for the potential biological activity of HPIDF and provide a theoretical basis for improving the biological activity of HPIDF. This work will also be the basic theory to functional research of Okara-HPIDF, which is also the core data of the relationship between the intake of Okara-HPIDF and colon health.

## 2. Materials and Methods

### 2.1. Preparation of Okara-HPIDF

Low purity dietary fiber from okara (okara-LPDF) was purchased from Shandong Sinoglory Health Food Co., Ltd., Liaocheng, China. After identification, its main components include: dietary fiber ≥ 60%, protein ≤ 26%, moisture ≤ 10%, ash ≤ 6%. HPIDF from okara was prepared by complex enzymatic method of LPDF [17]. The purity of HPIDF was 91.25% (according to the enzymatic-gravimetric method described in Chinese national standard GB 5009.88-2014).

### 2.2. The Fermentation of Okara-HPIDF

The preparation method of simulated intestinal fluid (SIF) was used following the United States Pharmacopeia (USP) [19]. Enzyme-free SIF was prepared in a sterile environment and sterilized at 121 ℃ for 20 min.

The collection method of feces was similar to the previous study [16] with minor changes: 10 g of feces was collected from C57BL/6 female mice (Beijing Vital River Laboratory Animal Technology Co. Ltd., Beijing, China), which was dissolved in 50 mL phosphate buffer solution (0.1 mol/L, pH = 6.5). After complete mixing, the residue was removed via filtration with four layers of gauze. The suspension was stored at −20 ℃ in a sterile centrifuge (finished in 1 h).

After the preparation of SIF and the extraction of colonic microorganisms, they were mixed in a ratio of 7:2 (*v*/*v*). The simulated intestinal fluid with a colonic microorganism (SIF-CM) was used as the fermentation environment for HPIDF. The SIF-CM solution was prepared just before use.

### 2.3. Bacterial 16S rDNA Sequencing

Feces collected in 2.2 were used for 16S rDNA sequencing. DNA extraction from feces was carried out using the QIAamp^®^ DNA Stool Mini Kit (QIAGEN, Germany) according to the manufacturer’s instructions. The quality of isolated DNA was evaluated on agarose gel electrophoresis and then DNA concentration was precisely measured using a NanoDrop NC2000 spectrophotometer (Thermo Fisher, Waltham, MA, USA).

The V3-V4 region of the 16S rRNA gene was amplified from the purified DNA of each sample. The primers used for the PCR amplification are as follows: the forward primer 515F (5′-ACTCCTACGGGAGGCAGCA-3′) and the reverse primer 907R (5′-GGACTACHVGGGTWTCTAAT-3’). The concentrations of PCR products were measured by the Quant-iT PicoGreen dsDNA Assay Kit (Invitrogen, Carlsbad, CA, USA). Afterward, all PCR products were pooled in equal amounts, and paired-end sequencing was performed using the Illumina MiSeq platform operated by Shanghai Personal Biotechnology Co., Ltd. (Shanghai, China).

### 2.4. Simulated Fermentation of Okara-HPIDF In Vitro

After mixing SIF-CM and HPIDF (9:1, *v*/*m*) under sterile conditions, the samples were cultured under anaerobic conditions at 37 ℃ for 16 h. After fermentation, the samples were filtered, the IDF was taken to determine the contents of the composition of monosaccharide after sterilization. The fermented-HPIDF (F-HPIDF) was used for the follow-up experiments after sterilization and freeze-drying.

### 2.5. Structure of Okara-HPIDF before/after Fermentation

#### 2.5.1. Scanning Electron Microscopy (SEM)

Merlin Compact scanning electron microscope (SEM; Carl Zeiss Jena GmbH, Jena, Germany) was used to observed the microstructure of HPIDF/F-HPIDF after the spray gold treatment. The scanning images were captured at accelerating voltages of 5.00 kV. All images were recorded at magnifications of 300× (low magnification) and 3000× (high magnification).

#### 2.5.2. Fourier Infrared Spectrum (FT-IR)

The polysaccharide functional group composition of HPIDF/F-HPIDF were performed using a Nicolet iS5 FT-IR spectrometer (Thermo Fisher, Waltham, MA, USA). The spectra were read over the range of 4000–400 cm^−1^ with a resolution of 4 cm^−1^ after the samples were mixed with KBr (1:100, *w*/*w*)

#### 2.5.3. X-ray Diffraction (XRD)

X-ray diffraction (XRD) analysis of the HPIDF/F-HPIDF was conducted according to the previous method [20], with some slight modifications. The diffraction patterns were recorded using a Rigaku Ultima IV (X-ray, Rigaku Corporation, Tokyo, Japan) operating at a voltage of 40 kV, an incident current of 40 mA, an anti-diffusion slit of 2/3, with Cu-Ka radiation (1 μ = 0.154 nm), and a scan speed of 1π/min in the range of diffraction angle from 10 to 80°.

#### 2.5.4. Particle Size and Specific Surface Area

The BT-9300HT laser particle sizer (Bettersize Instruments Ltd., Dandong, China) was used to analyze the particle size and specific surface area of HPIDF/F-HPIDF in which the concentration of the suspension is 4% (*m*/*v*).

### 2.6. Monosaccharide Composition of Okara-HPIDF before/after Fermentation and the Hydrolyzed

The monosaccharide compositions of HPIDF/F-HPIDF referred to the methods of the preliminary study [16]. The monosaccharide composition of the hydrolyzed was analyzed after freeze-drying by PMP pre-column derivatization and LC-10ATvp & SPD-10AVD HPLC system (Shimadzu, Tokyo, Japan) [21].

### 2.7. Adsorption Capacity in the Colon of Okara-HPIDF before/after Fermentation

#### 2.7.1. Basic Characteristics of HPIDF/F-HPIDF

We chose water-holding capacity (WHC), water-swelling capacity (WSC), and oil-holding capacity (OHC) as the basic characteristics of HPIDF/F-HPIDF. The determination was described by Zhang et al. [22] with minor changes. Briefly, water-holding capacity (WHC): 1.00 g sample (M1) and 20 mL of water were mixed in a dry centrifuge tube (M0). The sample was kept at RT for 24 h and centrifuged at 4000 rpm for 20 min. The supernatant was removed, and the weight (M2) was measured.

WHC (g/g) = (M2 − M0)/M1 * 100%

Water-swelling capacity (WSC): 0.10 g (M0) sample was taken into the calibration tube (V0), 10 mL water was added and let to stand for 24 h, and the volume (V1) was recorded.

WSC (mL/g) = (V1 − V0)/M0

Oil-holding capacity (OHC): 1.00 g sample (M1) and 20 mL of oil (soybean oil/lard) were mixed in a dry centrifuge tube (M0). The sample was kept at RT for 18 h and centrifuged at 4000 rpm for 20 min. The supernatant was removed, and the weight (M2) was measured.

OHC (g/g) = (M2 − M0)/M1 * 100%

Soybean oil represents unsaturated oil, and lard represents saturated oil.

#### 2.7.2. Heavy Metals-Adsorption Capacity (HMAC)

HMAC was measured by the methods of the previous study [16] with minor changes. The 50 μg/mL standard solution of Pb^2+^(Pb(NO_3_)_2_), Zn^2+^(Zn(NO_3_)_2_), Cu^2+^(Cu(NO_3_)_2_), and Cd^2+^(Cd(NO_3_)_2_) were prepared as the initial concentration to determine the HMAC of HPIDF/F-HPIDF. Total of 10 mL of four kinds of standard solutions were mixed with 0.1 g HPIDF (500 μg of metal ions in the system) separately, and stirred at 37 ℃ for 14 h before centrifuging at 4000 rpm for 20 min. The 240 Duo GFAAS (Agilent, Lexington, MA, USA) was used to detect the content of Pb^2+^, Zn^2+^, Cu^2+^, and Cd^2+^ in the supernatant according to the preinstalled methods of the workstation.

#### 2.7.3. Potentially Harmful Substances-Adsorption Capacity

Glucose-adsorption capacity (GAC), cholesterol-adsorption capacity (CAC), sodium cholate-adsorption capacity (SCAC), acrylamide-adsorption capacity (AAC), and nitrite-adsorption capacity (NAC) were selected as the measurement indicators of potentially harmful substances-adsorption capacity. The details are as follows:

1. GAC 0.1 g HPIDF/F-HPIDF, mixed with 10 mL 100 mmol/L glucose solution at 37 ℃ for 16 h, centrifuged at 4000 rpm for 20 min, and the supernatant was taken to determine the glucose concentration using a glucose kit (hexokinase method, A154-2-1, Nanjing Jiancheng Bioengineering Institute).
2. CAC 1 mg/mL cholesterol in ethanol solution was prepared. About 0.2 g HPIDF/F-HPIDF was mixed in 10 mL cholesterol solution. The adsorption and centrifugation conditions were the same as the above. Total cholesterol assay kit (A111-1-1, Nanjing Jiancheng Bioengineering Institute) was used to determine the cholesterol content in the supernatant.
3. SCAC 0.1 g HPIDF/F-HPIDF was mixed with 10 mL sodium cholate standard solution (0.2 g sodium cholate + 15 mmol/L NaCl aq 100 mL). The adsorption and centrifugation conditions were the same as 2.7.3-1. Furfural-sulfuric acid process [23] was used to determine the content of sodium cholate in the supernatant.
4. AAC 0.5 g HPIDF/F-HPIDF was mixed with 50 mL 15 mmol/L acrylamide solution. The adsorption and centrifugation conditions were the same as 2.7.3-1. HPLC was used to determine the concentration of acrylamide in the supernatant according to the method described in GB 5009.204-2014 by 1260 HPLC (Agilent, Lexington, MA, USA).
5. NAC 0.5 g HPIDF/F-HPIDF was mixed with 50 mL 100 mmol/L nitrite (Sodium nitrite) solution. The adsorption and centrifugation conditions were the same as the above. Ultraviolet spectrophotometry method (described in GB 5009.23-2010) was used to determine the concentration of sodium nitrite in the supernatant.

### 2.8. Statistical Analysis

The measurements of all samples should be repeated for at least three times and the results were expressed as the mean ± standard deviations ($\bar{x}$ ± SD). ANOVA with Duncan’s test and Tukey’s test was used to compare the data for the differences, *p* < 0.05 represented significant differences.MS Word and GraphPad Prism 6 (GraphPad Software Inc., San Diego, CA, USA) were used to organize the data, draw the graphs and tables.

## 3. Results and Discussion

### 3.1. The Gut Microbiota Structure in the Feces from C57BL/6 Mice

**Figure 1:**
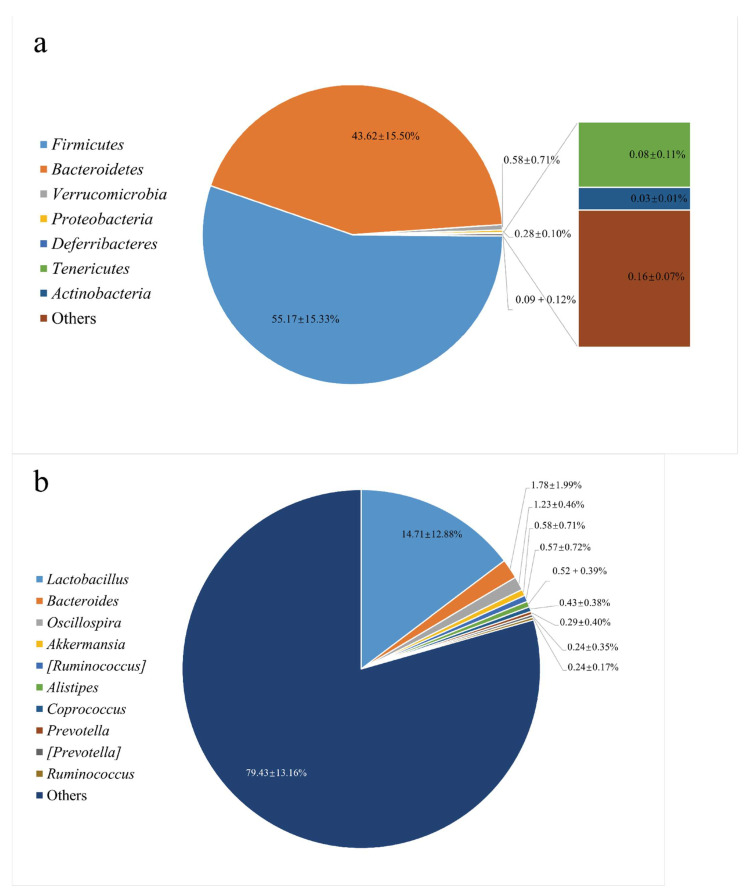
The gut microbiota structure in the feces from C57BL/6 mice. (**a**: Phylum Level; **b**: Genus Level).

The microbiota structure in the colon was analyzed by high-throughput sequencing spanning the 16S rDNA V3-V4 hypervariable region and shown in Figure 1. At the phylum level, *Firmicutes* (55.17%) and *Bacteroidetes* (43.62%) were abundant. As for the genus level, *Lactobacillus* (14.71%) accounted for the highest abundance of recognizable conventional microorganisms, which was consistent with the microbial composition of normal C57BL/6 mice feces [24]. C57BL/6 mice were used as a suitable animal model to evaluate dietary fiber and colon health. In other studies of HPIDF, we also chose this animal model. Therefore, we selected their feces for gut microbiota isolation and fermented HPIDF in vitro.

### 3.2. The Structural Changes of HPIDF after Being Fermented

#### 3.2.1. Scanning Electron Micrograph (SEM)

**Figure 2:**
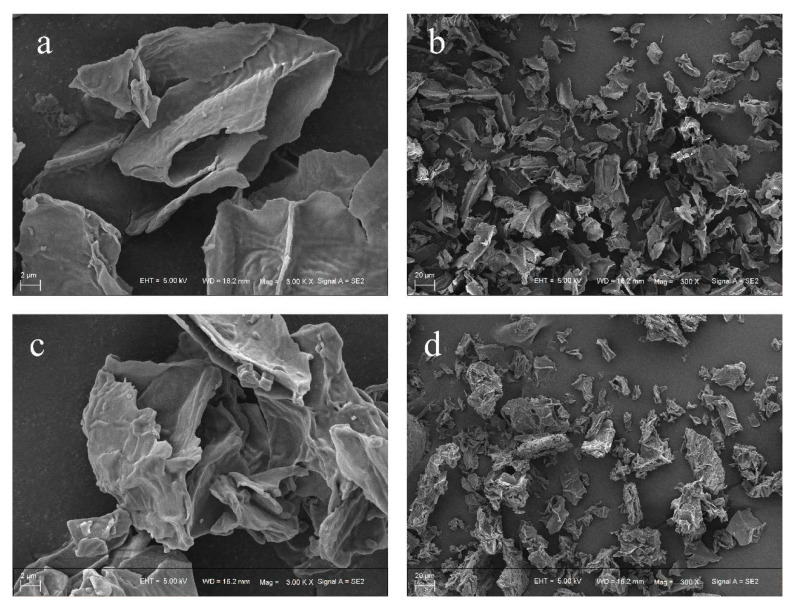
Scanning electron micrograph (SEM) of HPIDF/F-HPIDF. (**a**: HPIDF at high magnification; **b**: HPIDF at low magnification; **c**: F-HPIDF at high magnification; **d**: F-HPIDF at low magnification).

The microstructure of HPIDF/F-HPIDF at 300 times and 3000 times was shown in Figure 2. It can be observed that whether at low magnification or high, F-HPIDF showed a few differences compared to HPIDF. At low magnification (Figure 2a,c), the state of a single dietary fiber particle of F-HPIDF was richer in spatial structure and less smooth than that of HPIDF. In addition, the edge of F-HPIDF’s particle was smoother and the tearing feeling was lower than that of HPIDF. At high magnification (Figure 2b,d), the flatness of HPIDF was much higher than that of F-HPIDF, whose structure was chaotic and uneven. This might indicate that F-HPIDF had a higher specific surface area than HPIDF and may be attributed to the removal of some saccharide structure (monosaccharide, etc.,) and the fracture of fiber structure of HPIDF during fermentation. Therefore, we can speculate that some adsorption properties of F-HPIDF should be better than HPIDF because its specific surface area increased (Premise: no functional groups with adsorption properties were removed). This assumption needs to be verified by the following experiments.

#### 3.2.2. X-ray Diffraction (XRD)

**Figure 3:**
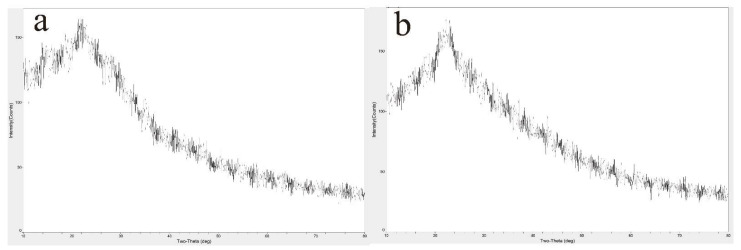
X-ray diffraction (XRD) of HPIDF/F-HPIDF. (**a**: HPIDF; **b**: F-HPIDF).

The XRD analysis of HPIDF/F-HPIDF is shown in Figure 3. It could be seen from the XRD spectra that, whether HPIDF or F-HPIDF, there was just one obvious crystal diffraction peak at 2θ = 22°, which was a characteristic XRD curve of hemicellulose [25]. Meanwhile, there was little difference between HPIDF and F-HPIDF, which proved our previous speculation that the cellulose content in HPIDF is less [16]. XRD spectra showed the same results. In short, the fermentation process did not change the crystal structure of HPIDF.

#### 3.2.3. Fourier Transform Infrared Spectroscopy (FT-IR)

**Figure 4:**
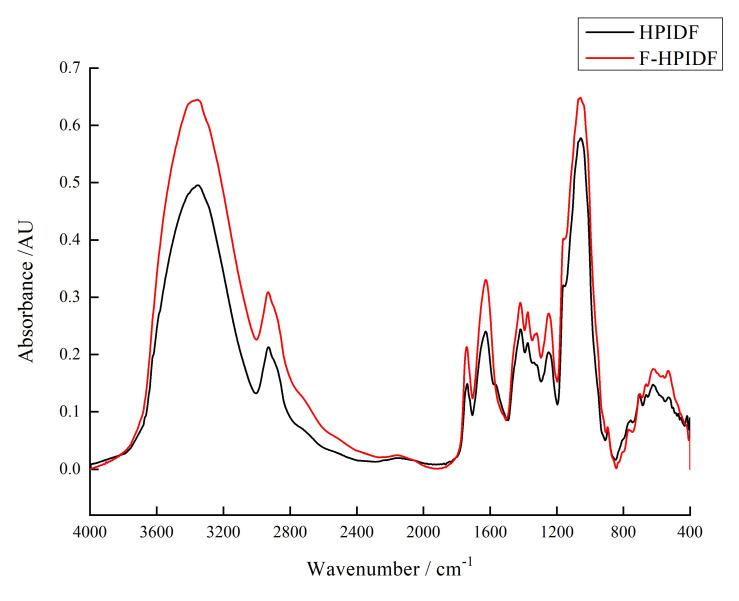
Fourier transform infrared spectrum (FTIR) of HPIDF/F-HPIDF. (Black: HPIDF; Red: F-HPIDF).

The FR-IR spectrum of HPIDF/F-HPIDF is shown in Figure 4. At 3353 cm^−1^, 2932 cm^−1^, 1737 cm^−1^, 1626 cm^−1^, 1418 cm^−1^, 1374 cm^−1^,1248 cm^−1^, 1057 cm^−1^, 706 cm^−1^, 623 cm^−1^, and 532 cm^−1^, both HPIDF/F-HPIDF showed significant absorption peak, which represented the composition of their polysaccharide functional groups. Actually, although HPIDF and F-HPIDF showed some differences in peak height, no differences in peak location were observed. In contrast with other studies [26,27,28], these absorption peaks represented O-H group in cellulose or hemicellulose, C-H stretching of -CH_3_ or =CH_2_ on carboxymethyl and methylene, oxygen (CO-OR) stretching vibration in hemicelluloses, C-O stretching vibration in the guaiacyl unit of lignin, glycuronic acid, etc. The other small peaks were not different from the previous study [16].

Although no differences were found in peak location, F-HPIDF had a markable characteristic in the FT-IR spectrum analysis. All peaks in F-HPIDF are higher than that in HPIDF, which means the fermentation increased the exposure of all functional groups. This result was consistent with the SEM results. The more complex the spatial structure, the greater the exposure of functional groups. This inference needs to be supported by the results of particle size and the specific surface area below.

To sum up, the fermentation might change the structure of HPIDF but did not change the composition, which would make the adsorption of F-HPIDF better than that of HPIDF, so that it may have better potential bioactivity in the colon.

#### 3.2.4. Particle Size and Specific Surface Area

Particle size and specific surface area of HPIDF/F-HPIDF are shown in Table 1,which both showed significant differences (*p* < 0.05). The smaller average particle size and the increase of specific surface area might lead to a higher adsorption capacity of harmful substances and metal ions. This result was in high agreement with the results of SEM and FTIR. These showed that the fermentation had made a great change in the structure of HPIDF. As for the composition, the analysis of monosaccharide composition of HPIDF, F-HPIDF, and hydrolysate was necessary.

### 3.3. The Monosaccharide Composition of HPIDF, F-HPIDF, and Hydrolysate

**Figure 5:**
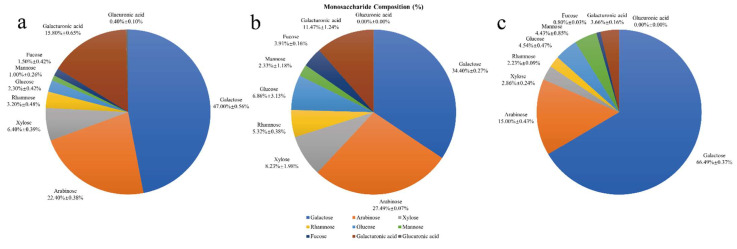
The monosaccharide composition of HPIDF, F-HPIDF, and hydrolysate. (**a**: HPIDF; **b**: F-HPIDF; **c**: Hydrolysate).

The monosaccharide composition of HPIDF, F-HPIDF, and hydrolysate is shown in Figure 5. The main constituents of HPIDF are shown in Figure 5a, which are same as the previous result [16]. With the progress of colonic fermentation, the monosaccharide composition of HPIDF changed significantly. F-HPIDF (Figure 5b) consisted of a less content of galactose (34.40%) and galacturonic acid (11.47%), meanwhile a higher content of arabinose (27.49%), rhamnose (5.32%), glucose (6.86%), and fucose (3.91%) than HPIDF. This showed that galactose and galacturonic acid were used as the carbon source of bacteria in the colon during fermentation.

As for the hydrolysate (Figure 5c), galactose (66.49%) and arabinose (15.00%) were the main components. The largest component in the hydrolysate should be considered as the monosaccharide that constituted the main component of HPIDF. As know, monosaccharides that make up hemicelluloses are mainly glucose, xylose, mannose, arabinose, and galactose [29]. This result proved that HPIDF is a dietary fiber with hemicellulose as the core. Then, during colonic fermentation, a large amount of hemicellulose was decomposed to free monosaccharides, which is a typical fermentation mode of hemicellulose [30].

The colonic fermentation model of HPIDF with hemicellulose as the main part should be regarded as one of the important findings of this study, which could help us to carry out more accurate research on the metabolic model of colonic microorganisms using HPIDF. On this basis, the metabolic pathway of free monosaccharides in the colon can be studied to make sure if they had any potential functional value. Even the modification of HPIDF could be carried out based on the result, at least we can add modifiers to the functional groups of hemicellulose so that to make these components easier to release in the colon.

In this experiment, the microorganism used to ferment HPIDF was mainly *Lactobacillus* (genus level), and the main components of HPIDF were hemicellulose and lignin. The fermentation mode of hemicellulose by *Lactobacillus* is relatively clear. In short, it is a process of using hemicellulose as the carbon source to produce lactic acid in the colon [31,32]. An increase in lactic acid, as a precursor of propionic acid [33], results in an increase of short-chain fatty acid (SCFA). This phenomenon is beneficial to colon health and may even prevent colon cancer [34,35]. In contrast, the high level of lactic acid in the colon may also have adverse effects on intestinal health [36]. However, we believed that the lactic acid produced by dietary fiber fermentation should not pose this risk because they are ingested non-exogenously.

As the above process occurred, many new monosaccharides would appear in the colon, in which the composition was mainly galactose and arabinose. Galactose is a widely recognized functional monosaccharide with biological activities, such as being conducive to the appreciation of *Bifidobacterium* [37]. Galactose binding to lectin [38], a process that occurs in the colon before cancerization, proved that galactose plays an important role in intestinal health. Arabinose is also an intestinal prebiotic, which can prevent or even treat colitis by affecting the composition of intestinal flora directly [39]. In short, the monosaccharides produced by the fermentation of colonic flora would have a positive effect on intestinal health.

### 3.4. The Adsorption Capacity of HPIDF/F-HPIDF

#### 3.4.1. WHC, WSC, and OHC of HPIDF/F-HPIDF

**Figure 6:**
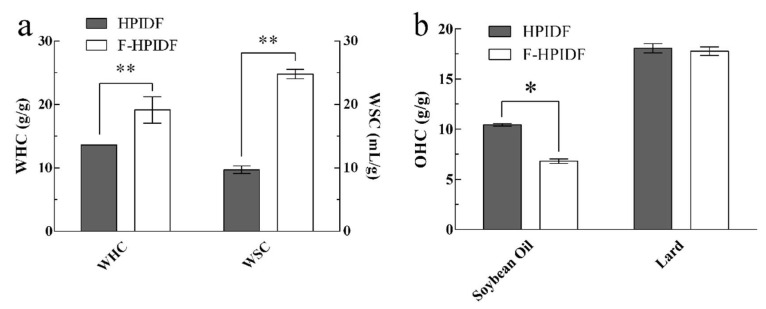
WHC, WSC and OHC of HPIDF/F-HPIDF. (**a**: WHC & WSC; **b**: OHC; *: *p* < 0.05; **: *p* < 0.01).

The WHC and WSC of HPIDF/F-HPIDF are shown in Figure 6a. Although the WHC and WSC of HPIDF are much higher than other IDFs [40,41], those of F-HPIDF (19.17 g/g, 24.83 mL/g) were still higher than HPIDF (*p* < 0.01). Carboxyl (-COOH) and hydroxyl (-OH) are the common polar functional groups in IDFs. The larger specific surface area and smaller particle size represented more polar groups in contact with the environment. The excellent water absorption of F-HPIDF was consistent with the previous results of the structure changes.

The OHC of HPIDF/F-HPIDF is shown in Figure 6b. The mechanisms of dietary fiber to adsorb saturated and unsaturated oils are different. So, we chose soybean oil and lard to determine the different OHCs of HPIDF/F-HPIDF. The result shows that the OHC of F-HPIDF for unsaturated fat (6.82 g/g) was lower than that of HPIDF (*p* < 0.05), however not different (17.77 g/g) from that for saturated fat (*p* > 0.05). Common hydrophobic groups include C-H stretching (-CH_3_ or =CH_2_), ethers (C-O), etc. This just showed that the hydrophobic groups in HPIDF were exposed less with the progress of fermentation, which might be attributed to the decomposition of hemicellulose during the progress described in 3.3. Acetyl should be considered as the characteristic functional group of hemicellulose [42], the decomposition of hemicellulose might lead to the loss of acetyl, which could be the result of the decrease of OHC. In contrast, saturated fat (such as lard) does not contain unsaturated bonds, which leads to a weaker dependence on acetyl than saturated fat. To prevent excessive absorption of fat by the intestine, a higher OHC is desirable. According to the fermentation mode of HPIDF in this study, we may try to modify the non-hemicellulose components so that they will not be decomposed by the gut flora while maintaining a high content of hydrophobic groups.

#### 3.4.2. HMAC of HPIDF/F-HPIDF

**Figure 7:**
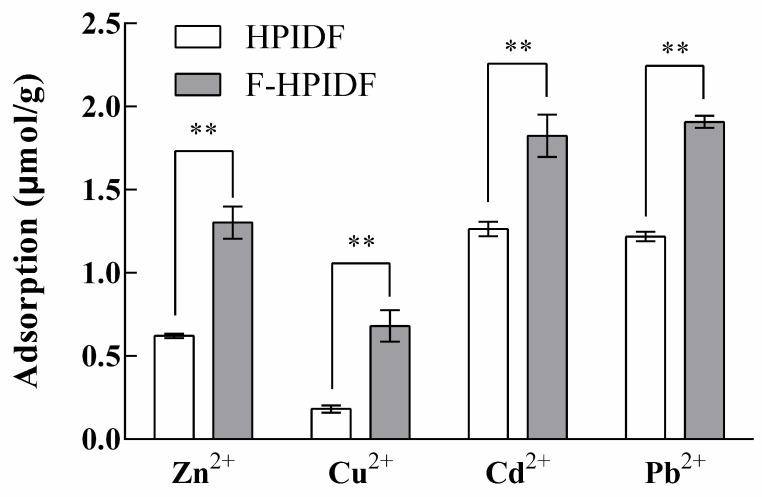
Heavy metal-adsorption capacity of HPIDF/F-HPIDF. (**: *p* < 0.01).

The adsorption capacity of HPIDF/F-HPIDF for metal ions is shown in Figure 7. On the whole, the adsorption capacities of F-HPIDF to four kinds of heavy metal were higher than that of HPIDF. The adsorption capacity of F-HPIDF for Cd^2+^ (1.82 μmol/g) and Pb^2+^ (1.91 μmol/g) was similar; meanwhile, the adsorption capacity of Cu^2+^ (0.68 μmol/g) was still the lowest. The HMAC of dietary fiber was directly related to its particle size and specific surface area [43], which led to the phenomenon in this study. This result was consistent with the structural analysis results.

The excessive intake of heavy metals was not only harmful to intestinal health but also might damage multiple organs [44,45]. With the progress of fermentation, the HMAC of HPIDF gradually became stronger, which was a very good situation for the digestive system. Heavy metal ions were difficult to enter the blood and would be excreted via feces. In short, the fermentation process of HPIDF in the colon may prevent the body from ingesting excessive heavy metals.

#### 3.4.3. Potentially Harmful Substances-Adsorption Capacity of HPIDF/F-HPIDF

**Figure 8:**
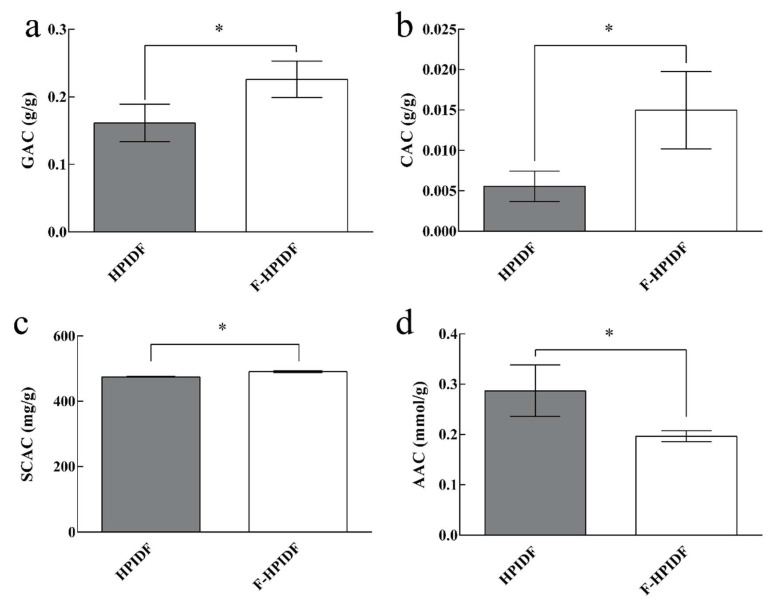
Potentially harmful substances-adsorption capacity of HPIDF/F-HPIDF. (**a**: GAC; **b**: CAC; **c**: SCAC; **d**: AAC; *: *p* < 0.05).

We used glucose-adsorption capacity, cholesterol-adsorption capacity, sodium cholate-adsorption capacity, acrylamide-adsorption capacity, and nitrite-adsorption capacity to determine the adsorption function to potentially harmful substances of HPIDF and F-HPIDF. The results are shown in Figure 8.

First, the adsorption capacity of HPIDF/F-HPIDF to nitrite was not shown in the results. According to previous research, HPIDF showed nitrite-adsorption capacity only in the gastric juice [16]. The same situation occurred in this study, we even did not find that F-HPIDF had a detectable nitrite-adsorption capacity. Therefore, the result was not shown in the figure. In short, whether it was fermented or not, HPIDF had no nitrite-adsorption capacity in the colon.

Normal amount of glucose is an important energy source for the body, but excessive glucose will cause a burden, which should be called a substance that may affect the health of the body. The GAC of F-HPIDF (Figure 8a) portrayed a better trend (0.23 g/g), suggesting that F-HPIDF may prevent glucose from being over absorbed in the intestine. The GAC of HPIDF is the result of multiple adsorption mechanisms [46]. Therefore, the higher available surface, smaller particle size, and more functional group exposure of F-HPIDF lead to better glucose adsorption than HPIDF. As one of the main places for the body to absorb glucose, the intestine could be prevented from absorbing due to the presence of F-HPIDF, especially the glucose produced by the decomposition of digestive juice.

As shown in Figure 8b, F-HPIDF (14.80 mg/g) exhibited a high cholesterol-adsorption capacity than HPIDF (5.58 mg/g). After F-HPIDF was mixed with cholesterol, the absorption in the colon of cholesterol could be controlled by reducing the solubility of cholesterol [47]. This mode of action was similar to GAC.

The SCAC of HPIDF/F-HPIDF is shown in Figure 8c. F-HPIDF (0.49 g/g) exhibited a small increase in SCAC than HPIDF (0.47 g/g). As a substance produced by bile acids, sodium cholate may lead to intestinal inflammation and even apoptosis [48,49]. A good sodium cholate-adsorption capacity may be a potentially beneficial effect on intestine health. Compared with our previous research [16], the sodium cholate-adsorption capacity in this experiment had a great improvement. We speculated that the reason may be the difference between the adsorption time. In the early stage, when we carried out an adsorption test in the digestive solution, 4 h was used for adsorption, which was much shorter than 14 h. In this experiment, to distinguish the differences in adsorption capacity before and after fermentation, we selected a long time to simulate the retention of dietary fiber in the intestine [50]. This showed that the adsorption of sodium cholate was still going on after 4 h. Then, the cholate adsorption kinetics of F-HPIDF may be also an object worthy of further study.

The acrylamide-adsorption capacity of F-HPIDF (0.20 mmol/g) showed a little decrease than HPIDF (Figure 8d). We had emphasized that the AAC of okara-HPIDF did not change with the gastroenteric environments. Therefore, AAC should only be related to the structure of the dietary fiber. The acrylamide-adsorption capacity on plant DF is generally weak, meanwhile mainly depended on physical adsorption [51]. Then we speculated that the decrease of AAC of F-HPIDF still came from the degradation of hemicellulose, although the specific surface area of F-HPIDF increased with the progress of fermentation. Hemicellulose was used as an adsorption material or matrix of acrylamide in previous studies [52,53].

In summary, the adsorption capacity of HPIDF to potentially harmful substances changed significantly after fermentation by colonic flora. The differences of F-HPIDF and HPIDF in properties were due to the changes in their structure during fermentation in vivo. Therefore, this process depended on the changes caused by the specific metabolic mechanism of colonic microorganisms.

## 4. Conclusions

In this study, the changes in the structure (SEM, FT-IR, XRD, particle size, specific area, and monosaccharide composition) and the adsorption capacity (WHC, WSC, OHC, heavy metal-adsorption capacity, and harmful substances-adsorption capacity) were analyzed to measure the changes between F-HPIDF and HPIDF. The results suggested that after being fermented by colonic microorganisms, the structure and properties of okara-HPIDF changed greatly. These findings provide a more accurate analysis of HPIDF after entering the digestive system. Meanwhile, the excellent adsorption and physicochemical properties caused by structural changes are beneficial to colon health. The intake of HPIDF might increase the number of beneficial monosaccharides in the colon, which might improve the composition of SCFA and have a beneficial impact on the body. This study also speculated that HPIDF may be modified to enhance its biological activity after its fermentation mode had been identified. The above all proved that HPIDF may have more utilization value.

## Figures and Tables

**Table 1:** Particle size and specific surface area of HPIDF/F-HPIDF.

	D_10_ (μm)	D_25_ (μm)	D_50_ (μm)	D_75_ (μm)	Specific Surface Area (m^2^/kg)
HPIDF	8.52 ± 0.08 ^a^	24.59 ± 0.58 ^a^	53.33 ± 1.45 ^a^	102.24 ± 5.69 ^a^	90.24 ± 1.27 ^a^
F-HPIDF	6.02 ± 0.05 ^b^	11.25 ± 0.12 ^b^	30.00 ± 0.39 ^b^	64.60 ± 1.48 ^b^	135.75 ± 1.36 ^b^

Different lowercase letters indicate a significant difference (*p* < 0.05).

## Data Availability

Raw data can be provided by the corresponding author on request.
